# History and Current Status of Mediterranean Spotted Fever (MSF) in the Crimean Peninsula and Neighboring Regions along the Black Sea Coast

**DOI:** 10.3390/pathogens12091161

**Published:** 2023-09-14

**Authors:** Muniver T. Gafarova, Marina E. Eremeeva

**Affiliations:** 1 S.I. Georgievsky Medical Academy (Academic Unit), V.I. Vernadsky Crimean Federal University, 295051 Simferopol, Russia; 2 Jiann-Ping Hsu College of Public Health, Georgia Southern University, Statesboro, GA 30458, USA

**Keywords:** Mediterranean spotted fever, *Rickettsia conorii*, *Rhipicephalus sanguineus*, Black Sea

## Abstract

Mediterranean spotted fever (MSF) is a tick-borne rickettsiosis caused by *Rickettsia conorii* subspecies *conorii* and transmitted to humans by *Rhipicephalus sanguineus* ticks. The disease was first discovered in Tunisia in 1910 and was subsequently reported from other Mediterranean countries. The first cases of MSF in the former Soviet Union were detected in 1936 on the Crimean Peninsula. This review summarizes the historic information and main features of MSF in that region and contemporary surveillance and control efforts for this rickettsiosis. Current data pertinent to the epidemiology of the disease, circulation of the ticks and distribution of animal hosts are discussed and compared for each of the countries in the Black Sea basin where MSF occurs.

## 1. Introduction

Mediterranean spotted fever (MSF) is a tick-borne disease caused by *Rickettsia conorii* subsp. *conorii.* Conor and Bruch described this disease as a highly febrile illness with dermal spots (fièvre boutonneuse) that they observed in seven patients in Tunisia in 1910 [1]. In the following years, a similar disease was diagnosed in other regions surrounding the Mediterranean Sea, including Spain, Italy, Greece and the southern border of France. Interest expanded in 1925 after Olmer identified a cluster of similar cases in Marseille, France [2]. In 1927, Boinet and Pieri described the clinical hallmark of the illness, a “tache noire” or eschar, the skin lesion which develops at the site of an infected tick attachment [3]. Experimental work by Durand and Conseil and Blanc and Caminopetros demonstrated that the brown dog tick *Rhipicephalus sanguineus* serves both as the vector of the disease and reservoir of the agent [4,5]. The etiological agent of MSF was named *Rickettsia conorii* in 1932. With the most useful nomenclature of the *Rickettsia* species, *R. conorii* sensu stricto is called *R. conorii* subspecies *conorii* and it belongs to the *R. conorii* complex together with three other subspecies [6]. Strain Malish 7 is the reference type isolate which defines the prototypical characteristics of the etiological agent.

MSF is now known to be endemic in areas extending throughout the countries of the Mediterranean, Black and Caspian Seas; to Sub Saharan, Central and South Africa; the Middle East; the Indian subcontinent; and to China [7,8,9,10]. Depending on the region, the prevalence and clinical manifestations of the disease typically diagnosed as MSF vary substantially. This is largely because local (autochthonous) populations of ticks differ in their ecological associations and repertoire of circulating SFGR [11,12], and the co-occurrence of other subspecies of the *R. conorii* complex, including the *R. conorii* subspecies *israelensis*, *R. conorii* subspecies *caspia* and *R. conorii* subspecies *indica*, which can be transmitted and maintained by *Rhipicephalus* spp. ticks [13,14,15,16,17]. The varied genetic traits of people in these regions can also affect the presentation of these diseases [18,19,20].

The aim of this review is to examine the specific features and current trends of MSF in countries located in the Black Sea littoral, with special focus on the endemic situation, epidemiology and clinical features of MSF in the Crimean Peninsula. 

## 2. Historic Summary of MSF in Crimea

The first cases of MSF were identified in Crimea (city of Sevastopol) in 1936 during a parasitology expedition headed by A. Ya. Alymov [21]. These observations and subsequent work by Andreev produced a detailed description of the clinical illness and its epidemiological characteristics, which were consistent with earlier observations by French authors [22]. Most importantly, these clinical observations were corroborated by laboratory isolation of rickettsiae from the blood of a patient and ticks collected from the patient’s dogs [22]. Close contact with *Rhipicephalus sanguineus*-infested dogs was noted for each patient. Interesting details were noted in two instances: (1) a pediatric case occurring after a child removed ticks from a neighbor’s dogs and (2) a case in a man occurring after he slept with his dog during a hunting trip. Both cases resulted in the onset of MSF 5 days after these exposures. 

Subsequent passive surveillance and active investigations identified 27 new cases of MSF in other parts of Crimea, including Yalta, Evpatoria, Kerch, Dzhankoy and Simferopol, during 1936–1938. Eight cases of MSF were recorded by the Sevastopol Department of Infectious Diseases in 1936, and seven patients each in 1937 and 1938 [23]. 

No formal disease surveillance activities were conducted during and immediately after World War II, although according to anecdotal sources, numerous cases of MSF occurred during that period of time. In the following decade of 1947–1957, 52 cases of MSF were identified in Crimea [24,25]. 

During 1958–1960, a comprehensive campaign consisting of epidemiological surveillance, veterinary and environmental tick control and dog owner education was implemented to mitigate the occurrence of MSF in Sevastopol and its vicinity [25,26]. These concerted efforts resulted in almost complete elimination of cases of MSF, which were only rarely identified in areas designated as control sites for this campaign. This systematic approach to tick control was in place for almost 40 subsequent years and resulted in only sporadic cases of MSF, with an annual incidence ranging from 0.4 to 1.44 per 100 thousand population [24,26]. 

## 3. History of MSF in Other Regions of the Black Sea Basin (1931–1990s)

**Figure 1:**
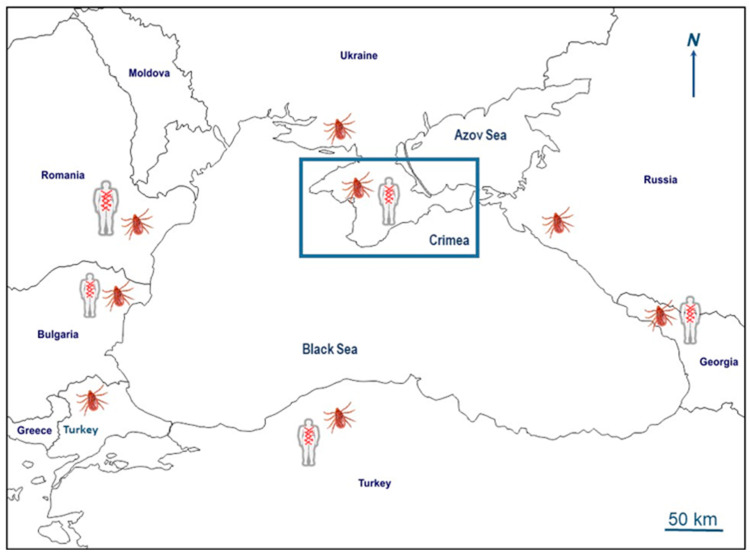
Map depicting the Black Sea coast and neighboring countries. Symbols indicate reported clinical cases diagnosed as MSF and detection and/or isolation of *Rickettsia conorii conorii* from *Rhipicephalus sanguineus* in corresponding regions. Figure was generated using counter map from https://d-maps.com/m/mediterranean/mernoire/mernoire10.gif (accessed on 25 June 2023).

The geographic locations of the countries with Black Sea littoral regions are depicted in Figure 1. 

In 1947, Zdrodovskii and Golinevich investigated the endemic foci of MSF on the Caucasian coast of the Black Sea (city of Sukhumi) and described the isolation of an *R. conorii* strain from *R. sanguineus* collected from a dog in a patient’s household [27]. The isolate had biological properties consistent with virulent strains of *Rickettsia*, as it caused 7 days of fever and periorchitis in guinea pigs and fever and lymphadenopathy in rhesus macaques. Clinical features of contemporary cases of MSF diagnosed along the Caucasus coast of the Black Sea were summarized by Avetisova [28]. 

In Romania, the first reported outbreak of boutonneuse fever occurred during the summer of 1931 in Constanta (Constanza district of the southern Romania area that includes the Danube delta and Black Sea coast); it involved 34 individuals [29,30]. MSF has been diagnosed in Romania since 1910; 11 cases were identified prior to 1930 and an additional 4 cases in 1932–1934 [30]. This is an interesting fact, since Romania is not a Mediterranean country. The disease was probably overlooked in the following years and during World War II until a 1948 outbreak in the capital city of Bucharest that continued until 1951 with 89 cases [30]. Both the 1931 and 1948 outbreaks were linked to the presence of dogs parasitized by *R. sanguineus*, and an SFG *Rickettsia* isolate was recovered from the blood of one of the patients from Bucharest [29,30]. In subsequent years through the end of the 20th century, only sporadic cases and community-centered outbreaks were reported (cited in [31]).

Recognition of the first clinical case of MSF in Bulgaria is attributed to the work conducted by I. Vaptzarov in 1948, followed by the subsequent isolation of a regional strain of *R. conorii* in 1956 by Shindarov (cited in [32]). This episode occurred in the Plovdiv area, the inland district of Bulgaria; however, MSF was subsequently recognized along the Black Sea coast, including the Varna and Burgas districts, as well as in other parts of the country. The dynamics of MSF are thought to have two waves in Bulgaria; the first started in 1948 and continued until 1970 with 240 total MSF cases reported, including sporadic cases and small group outbreaks (cited in [32]). No MSF cases were reported during 1970–1992, which was attributed to effective control of stray dogs, vector control and changes in animal farming and agriculture practices. The second wave of MSF in Bulgaria started in 1993 when an initial ten cases were confirmed using microimmunofluorescence; it reached over 10,000 cases by 2004, with the largest incidence occurring in the south-eastern and southern parts of the country, but with sporadic cases diagnosed across the country and outside the main endemic areas [32,33]. 

Turkey has an unusual geographic position, with its northern border touching the Black Sea and its southern and western borders connecting to the Mediterranean Sea. MSF has been described in Turkey since 1938; however, it was not considered to be a public health issue due to its low incidence, morbidity, and mortality (cited in [21,34]). Only sporadic cases were reported during the 1970s and 1980s. Cases of MSF diagnosed based on clinical and epidemiological features have been described in Bursa, Edirne and Istanbul since 1987 and, most recently, with the help of serological and molecular diagnostic methods in the Ankara, Antalya and Trakya regions [35,36,37,38]. The occurrence of MSF in the Trakya region and Ankara was confirmed via isolation of *Rickettsia conorii conorii* from skin and PCR detection of *R. conorii* DNA in the skin and blood of patients [37,39].

## 4. Epidemiological Status of MSF in Crimea and Other Countries in the Black Sea Region (1990–2021)

**Figure 2:**
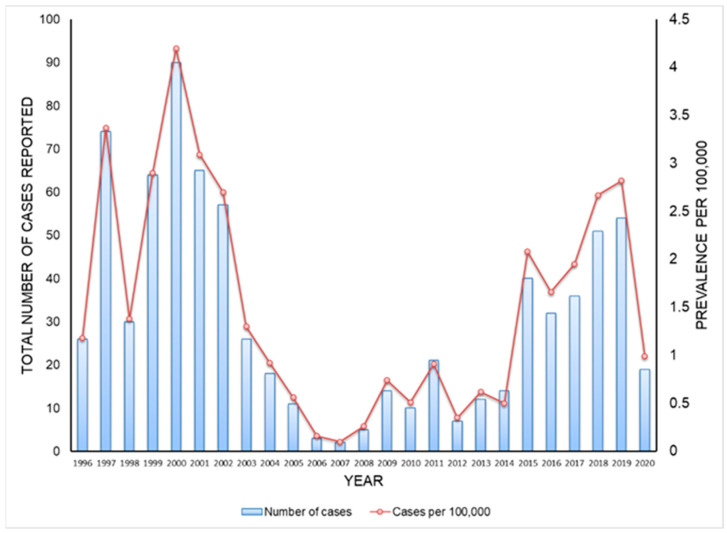
Incidence of MSF in Crimean Peninsula. Original data.

Since the 1990s, economic instability in the region and the lack of tick and veterinary surveillance and control efforts have led to a large increase in roaming stray dogs with massive tick infestations in Crimea. This situation caused the re-emergence of old and the emergence of new foci of MSF throughout the Crimean Peninsula which affected the cities of Evpatoria, Yalta, Alushta, Sudak, Feodosia, Kerch, Saki, Black Sea, Simferopol, Bakhchisarai, Leninsky and other districts. Available data about formally reported cases during the last 25 years of passive surveillance indicated a peak in cases occurred in 2000 followed by a deep plunge during 2003–2014 and an apparent continuous upswing until cases declined during the COVID-19 outbreak (Figure 2). This decline in reporting and diagnosis of tick-borne diseases during the COVID-19 epidemic has been reported in other countries worldwide [40,41,42]. 

Whether these numbers reflect the actual epidemiological situation and impact of MSF on the local population is not known; however, it is probable that the true prevalence of this infection in the region is largely underreported. This speculation is based on an 11.1% seroprevalence to the *R. sibirica* antigen detected using a complement fixation test among 350 healthy blood donors in Crimea [43]. To better understand these findings, it would require another serological survey using more state-of-the-art diagnostic tests such as ELISA, MIF and Western blotting and cross-absorption assays using multiple antigens from *Rickettsia* found in this region [44]. 

**Figure 3:**
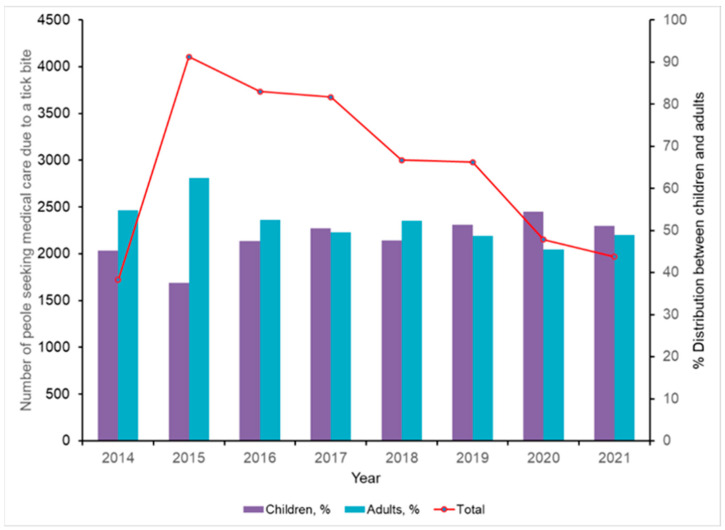
Known records of people seeking medical care due to illness after a tick bite in Crimea. Primary Y-axis indicates number of people seeking medical care and secondary Y-axis shows percent distribution among adult and pediatric patients. Original data.

The observed increase in the number of MSF cases in Crimea in recent decades is consistent with the large number of local residents seeking medical care following tick bites (Figure 3). Although it appears that there are no age-related differences in tick-bite incidence, there were statistically significant higher numbers of adult visits in 2014 and 2015 (*p* < 0.001), while in 2020 there were more records of tick attachment(s) to children. Most of these bites occurred during early May through the end of June. The incidence of travel cases of MSF in tourists visiting Crimea is not known, since those infections are not formally captured and, with few exceptions, are probably largely missed [45].

**Figure 4:**
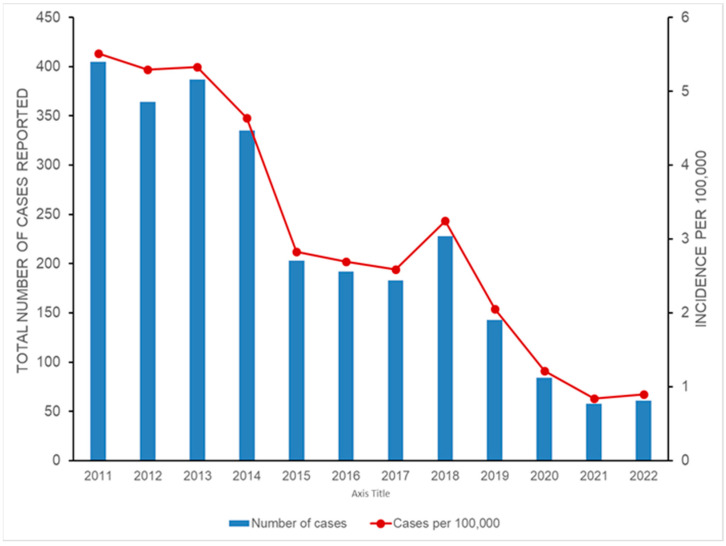
Incidence of MSF in Bulgaria. Data were obtained from the website of the National Center for Infectious and Parasitic Diseases, Sofia, Bulgaria [54]. Population estimates were obtained from [55].

MSF is considered to be a wide-spread rickettsisosis in 21st century Bulgaria and occurs along both the coastal and inland districts, with the highest incidence reported from south-eastern and southern areas of the country including the Burgas, Varna, Slivan, Stara Zagora, Haskovo, Jambol, Plovdiv and Pazardjik regions [32]. Early in the 21st century, the total annual number of reported cases of MSF exceeded 1500 and its incidence rate was higher than in endemic countries situated in the Mediterranean basin, like Croatia or Algeria; however, a continuous decline was detected during the following years, with the lowest incidence observed during the COVID-19 outbreak and 2021–2022 (Figure 4) [46]. MSF is diagnosed in all age groups, with milder clinical forms diagnosed in children and moderate to severe forms of illness in adults and especially elderly patients [47,48,49]. MSF cases occur from March throughout November with peak numbers in July–August. In contrast to cases during 1940–1950, urban residents are more frequently affected and contact with dogs and tick contact (removing, squashing and/or being bitten) are among the top risk factors [32,49,50]. The MSF fatality rate in Bulgaria varied depending on the region: it was reported to be 0.9% (n = 902) in the Pazardzhic region in 1996–2003, 1.47% (n = 885) in the Varna region among cases diagnosed during 1994–2003 and 3.46% (n = 774) during 1993–2003 in the Plovdiv region [49,50,51]; only one fatal MSF case was diagnosed in 2022. Both the occurrence of severe disease and lethal outcomes were associated with advanced age of the patient, underlying health conditions, delayed doctor visits and/or inadequate treatment and patient management; these are common issues with patients suffering from rickettsial diseases [49,52]. Lack of familiarity with MSF among physicians was another factor that complicated patient care at the onset of the second wave of MSF in Bulgaria; this is again a common problem affecting the proper diagnosis and management of rickettsioses [53]. 

According to the Romanian National Institute of Public Health (https://www.cnscbt.ro/, accessed on 26 June 2023), the highest incidence of 4.42 cases per 100,000 was in 2001 [56]. This situation mostly impacted two southern coastal districts, Constanta and Tulcea, which experienced 10-fold (in 2000) and 8-fold (in 2005) increases in MSF case numbers, respectively, compared with the rest of the country [56]. These regions remained the primary sites of MSF in Romania through 2016 (the last year surveillance data were available from the CNSCBT annual reports), although the overall country-level incidence rate decreased by 2005 and remains low at 0.3 cases per 100,000 [56]. According to the Romanian national surveillance system, 80% of reported MSF cases were confirmed via serologic testing of paired sera, and the remaining cases were classified as probable based on a single serum test result [31]. Both rural and urban residents become infected, and individuals aged 45 years and older are most often infected. Clinical manifestation is mild in 58% of cases, although two deaths were reported during 2000–2008 in Constanta [57]. MSF cases occurred from April through November with the highest incidence in May through August, consistent with the questing behavior of *R. sanguineus* [58,59,60]. Most patients reported tick exposure, or contacts with dogs and other tick-infested peridomestic animals, thus corroborating observations from other countries. 

Since MSF is not a reportable condition in Turkey, its incidence and morbidity may only be evaluated based on case reports. Clinical manifestations are very similar to those reported in other countries in the Black Sea basin [35,36,61], with pancytopenia being one atypical symptom [62]. Elderly people and individuals with concomitant hypertension or diabetes mellitus are at higher risk for severe illness and death [35]. MSF may be significantly under-recognized and underdiagnosed in Turkey due to the significant resemblance of its severe forms to severe sepsis [38] and viral hemorrhagic fevers, particularly Crimean Congo Hemorrhagic fever [35,61]. 

## 5. Description of Main Clinical Features of MSF in Crimea

The clinical manifestations of MSF in Crimea most often match descriptions of this disease in other endemic locations (Table 1). These consist of the onset of an acute febrile illness after a 5–10-day incubation period (post-tick exposure) with a body temperature increase to 39–40 °C which lasts for 3 to 10 days, headache, joint pain, development of an eschar (72% of patients) and regional lymphadenopathy (32%) [21,22,23]. A polymorphic, maculo-papular and papular-petechial rash, with spots 2–10 mm in diameter, develops on days 2 to 4 of illness and persists for up to 4 days. When first apparent, spots are pink, but eventually become dark red and even cyanotic in color in the later stage of the illness. The whole body is affected, including the development of a rash on the palms and soles in 80% of individuals. Typically, the disease course is relatively mild, but elderly individuals are most often severely affected and present with very high fever. According to the early observations, most patients do not develop classic “Typhus-like” neurological signs [21,22,23]. According to Alymov, patients recover in a few days after their temperature returns to normal [21], a remarkable outcome since these observations were made before antibiotics were used for the treatment of rickettsioses. The rash disappears starting on about day 7 of illness, but its traces can be seen up to day 75 after the onset of the disease and associated elevated pigmentation can be observed even longer in some individuals. Pigmentation developed at the site of the eschar can persist for 2–3 years after complete convalescence.

Recent clinical cases of MSF in Crimea often have more severe morbidity and moderate (83.2%) and severe (24.6%) courses of infection and more frequent occurrence of atypical clinical manifestations [63,64,65] (Table 1). The first formally recorded fatal cases due to *R. conorii* in Crimea were diagnosed in 1996 as a part of MSF outbreak investigation in the Saki region [66]; 31 cases were identified, including 2 fatalities involving elderly patients. Although access to modern laboratory diagnostic methods is generally not available for most clinical diagnostic labs, 7 out 14 Crimea MSF cases were confirmed with PCR in 2014, and all cases were mild to medium severity [67]. Only one severe illness was diagnosed among fourteen MSF cases reported in 2015 [67]. According to recent observations, a severe course of MSF and prolonged recovery are commonly observed among Crimean patients aged over 50 years with preexisting heart ischemia and a history of myocardial infarction, atrial fibrillation, heart failure and chronic obstructive bronchitis, as well as in individuals suffering from chronic alcoholism and homeless persons [63,64]. The significance of poor cardiac health and associated symptoms for poor outcomes from MSF was also noted by the original investigators of MSF in Crimea [21,22,23]; this correlation is consistent with the classic observations reported by Olmer [2].

## 6. Analysis of Recent Clinical Cases of MSF in Crimea

**Figure 5:**
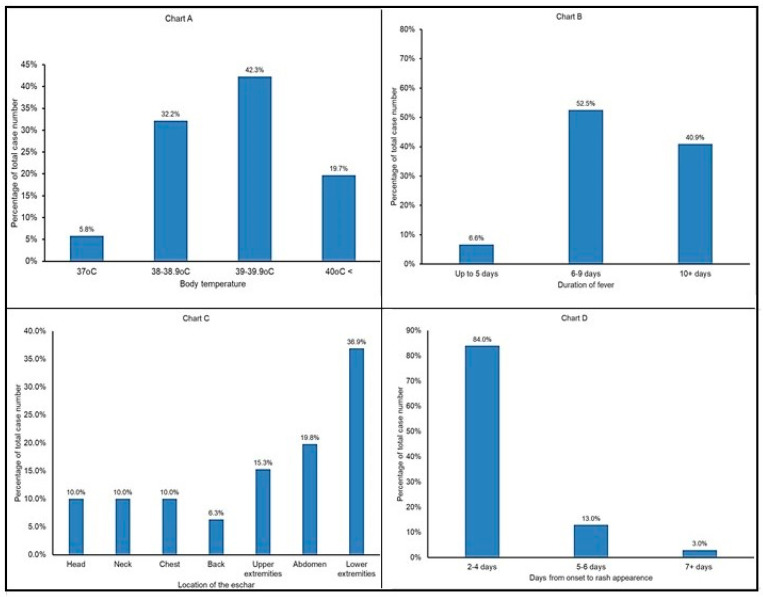
Summary of the clinical symptoms in recent cases of MSF in Crimea. (**A**) Frequency of body temperature, (**B**) duration of fever, (**C**) location of eschar, (**D**) days from disease onset to rash development.

A summary of the most recent MSF cases in Crimea identified five main stages of the illness, including the incubation period, pre-exanthema stage, exanthema and full-blown disease, followed by a convalescence period [63,64]. The incubation period lasts, on average, for 5–7 days (range from 2 to 15 days); in 27 individuals with good recall of finding and removal of ticks, the incubation period averaged 6.3 ± 0.2 days. The onset is acute and characterized by a sharp increase in the body temperature to 39–40 °C with chills in most patients (Figure 5A). In 75.2% of the patients, their temperature remained stable before therapy was started; 15.3% of the patients experienced only morning temperature spikes and 9.5% suffered from intermittent fever. Fever lasted between one and two weeks in most patients depending upon the time of admission to the hospital and start of the treatment (Figure 5B), and it decreased after 4–6 days of treatment.

Eighty-three percent of the patients had a headache which had a persisting diffuse character and was moderate in half of the individuals. In some patients, the headache radiated to the periorbital region, and was accompanied by dizziness, photophobia and sleep disturbance. Myalgia was observed in half of the patients; its manifestations varied from very intense, impeding movement, to moderate, with muscle weakness and aches throughout the body. One-third had arthralgias, predominantly symmetrical without dysfunction of the joints. In 25% of patients, arthralgia and myalgia appeared earlier in the joints and muscles closest to the tick attachment site, but it was especially pronounced when the eschar was in the lower extremities. Headache, myalgia and arthralgia resolved within 5–7 days of antibiotic, antipyretic and intravenous fluid therapy in most patients, although in approximately one-third of the patients, headache and arthralgia persisted for an additional 2–3 weeks following convalescence.

**Figure 6:**
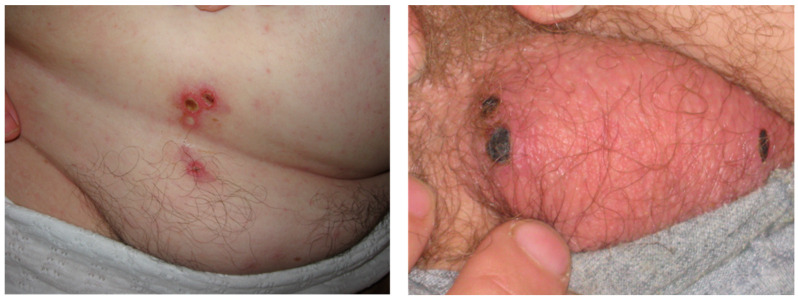
Multiple eschars on skin of a patient clinically diagnosed with Mediterranean spotted fever (Photo by M.T. Gafarova with patient consent).

An eschar was found in 81% of the patients; the lower extremities and abdominal areas were the most common locations of the eschar, followed by the upper extremities and upper parts of the body (Figure 5C); multiple eschars were present in some instances (Figure 6). In addition, unilateral catarrhal conjunctivitis was present in 1.8% of cases. A total of 23% of the patients with an eschar had regional lymphadenitis but only 2.2% patients had enlarged lymph nodes nearest to the eschar site. Inguinal lymph nodes of the corresponding side were enlarged when an eschar was present on the lower extremities or in the lower abdominal region. Enlarged axillary lymph nodes occurred when an eschar was present on the skin of the chest, upper back or upper limbs. Cervical and submandibular lymph node enlargement was detected when the eschar was on the scalp or neck. These observations were in good agreement with historic reports of MSF in Crimea [21,22,64,65].

The development of eschars occurred in several steps consistent with the stage of the disease. At admission to the hospital (on average, 6.3 ± 0.2 days of illness), it manifested as a superficial necrotic area of s kin of 0.5 to 2.0 cm in diameter covered with a thin hemorrhagic crust (scab) tightly welded to the underlying tissues; bleeding typically occurred if this scab was lifted. Necrosis in 105 (76.6%) patients was located on an infiltrated base, around which a zone of hyperemia was present in the form of a ring 1 to 5 cm in diameter. The eschar area was generally painless on palpation, but some patients reported subjective feelings of burning, itching and mild soreness. Typically, the scab would come off within 3–4 days following a temperature drop, and the exposed wound would epithelialize without a scar within 5–7 days. Ring-like skin peeling, and subsequent pigmentation would develop at this hyperemia area of the eschar and last at least for 2–3 months after convalescence.

**Figure 7:**
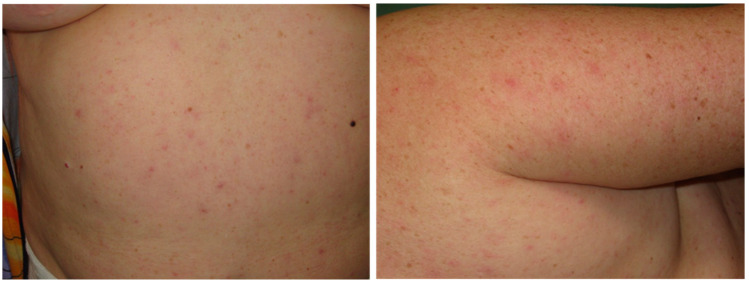
Maculopapular rash on the skin of patients clinically diagnosed with Mediterranean spotted fever (Photo by M.T. Gafarova with patient consents).

According to our observations, 96.3% of MSF patients had a rash which developed within 2–4 days following the onset of the illness (Figure 5D and Figure 7). Typically, it first appears on the body trunk and lower extremities, and then spreads to the rest of the body, including the face (78.8%) palms and soles (69%). At the peak of the disease, most patients had a puffy pale face (in 5% of cases face hyperemia was noted), shiny eyes, injected sclera and their skin was moist and hot to the touch. A combination of these symptoms was consistent with the clinical profile of MSF described earlier in Crimea [22]. Data presented in earlier studies also indicated that a rash develops in most cases; its prevalence varied from 100% in a 1952 study to 77.4–80% in 2009 [64,65].

In total, 73% of the patients had maculopapular rash, and roseola-papular rush was present in 23.3% of patients; the few remaining patients had no skin lesions. The rash first appears in the form of rosaceae, which then transforms within 1–2 days into macula and papules in 1–2 days. Their color varies from pale pink to brownish red with a cyanotic tint. Moderately abundant rash was observed in 40% of the patients and only single lesions were found in 10% of patients, typically localized on the exterior surfaces of the limbs, an observation consistent with the data of other authors [21,22,65,68]. At the peak of disease, 3.7% of patients had enanthema on the soft palate in the form of few petechia-like lesions. The rash started to resolve with a drop in temperature, on average after 4–5 days of antibiotic therapy. Individual lesions faded, decreased in size and the surface of the skin became smoother. Areas with petechiae and papules could remain pigmented for 3–4 weeks after convalescence.

Organ system involvement, particularly the respiratory and cardiovascular systems, was most remarkable at the peak of the illness and reflected changes due to interstitial inflammatory processes underlying progressive pathological changes due to rickettsia-caused vasculitis [69]. According to our observations, on admission, patients complained of a loss of appetite (58.3%), sore throat, nasal congestion, dry cough, shortness of breath, moderate chest pain (3.6%) and palpitations (1.5%). Hepatomegaly was diagnosed in 42.3% of patients. As the disease progressed, patients developed a bradycardia accompanied by hypotension in one-third of the cases. These symptoms are consistent with clinical manifestations of myocarditis and other cardio-vascular conditions developed as a part of disease progression [70,71]; however, they may be also the result of preexisting heart conditions in elderly patients and individuals with a history of ischemic heart disease and myocardial infarction. Different from the original reports of MSF in Crimea, 31.3% of patients suffered from bradypsychia (slow reaction) and bradilalia (speech was affected), but these conditions improved after 3–4 days of therapy. Severe forms of neurological disorder(s) were noted in two patients who had night-time hallucinations at the peak of the fever, and one individual experienced a short-term loss of consciousness which was interpreted as a manifestation of “typhoid status”.

## 7. Vector and Host Associations of *Rickettsia conorii* in Crimea and Other Countries in the Black Sea Region

The brown dog tick, *Rhipicephalus sanguineus* Latreille, is the vector and reservoir of *R. conorii conorii* [5]. Numerous studies were conducted in Crimea to determine the distribution and host associations of this tick [72,73,74,75,76,77]. In-depth study and evaluation of diverse species of wild and domestic animals identified dogs, *Canis familiaris*, as the main vertebrate host of *R. sanguineus*; however, this tick can feed on other animals [72,74,76]. According to studies from the 1960s conducted in Crimea, *R. sanguineus* was found mostly on dogs (abundance index [number of ticks per animal] up to 114.0), but it was present infrequently on cows (0.05–1.7), sheep (0.17) and goats (0.1) [74]. This tick will also infest cats, foxes and other wild canids [72].

The life cycle of *Rh. sanguineus* consists of four stages—eggs and three active parasitic stages (larva, nymph and the adults, male and female) that require a bloodmeal [11,12]. *Rh. sanguineus* is a one-host tick feeding on one species of animal in the larval, nymphal and adult stages; gravid females leave the host for the ground prior to laying eggs. Once on the ground, the female starts oviposition in 7–10 days, and larvae will hatch after 36–47 days. Larvae will molt into nymphs 7–9 days following a bloodmeal, and nymphs will molt into adults 12–13 days after engorgement. The entire life cycle lasts 3 months, although these events may be accelerated or delayed depending on environmental temperature and humidity [11,12]. In Crimea, adult *Rh. sanguineus* are typically found on dogs from March to September, with peak infestations observed in May–June and August–September [74,76]; nymphs are rare in April and abundant in July–August. *Rh. sanguineus* can overwinter as larvae, nymphs or adults. This tick can also establish persisting in-house infestations and survive in internal and external crevices [78,79,80]; however, this type of behavior has not been reported in Crimea.

Naïve *Rh. sanguineus* can acquire *R. conorii conorii* at any life stage as a part of the bloodmeal when feeding on a rickettsiemic dog or another animal, or from another tick through co-feeding [12,81]. Infected ticks will transmit rickettsiae to a naïve animal during feeding. Dogs are highly susceptible to *R. conorii* and remain infectious to ticks for at least a month post-infection [81]; however, poor breeds are more prone to rickettsial infection and differ in severity of *R. conorii* infection [82].

Once a tick is infected, *R. conorii* establishes a systemic infection and is transmitted transtadially and transovarially; however, the net effect on the infected tick is detrimental [83]. The exposure of *Rh. sanguineus* to *R. conorii conorii* strain Malish during experimental feeding infection produced lasting harmful effects on their survival [84,85], including a reduction in engorged larva-to-nymph molting success and a decreased survival in questing nymphs. Furthermore, the *R. conorii* infection rate was low in surviving ticks. However, significant variations were reported pertinent to the degree of damage caused by different *R. conorii* subspecies [86,87]; these phenomena may reflect unique properties of various *Rickettsia* isolates and how well they propagate in the ovary of a particular tick [88].

Historically, other tick species have also been identified as vectors of *R. conorii* [7,8]; however, retrospectively, many of these associations may be misleading because they were established before molecular studies became widely available for ecological and epidemiological investigations of individual rickettsial agents and tick population genetics. In this regard, the difficulties inherent in identifying various genotypes of *Rhipicephalus sanguineus* sensu lato should be emphasized [89], as well as complications in accurately speciating engorged immature ticks and interpreting the test results [90]. In African countries, *R. conorii conorii* has been detected in *Rhipicephalus simus* and *Haemaphysalis leachi* [91].

## 8. Contemporary Tick Surveillance Efforts in Countries Adjacent to the Black Sea

Contemporary acarological studies conducted in the Crimea Peninsula during the last decade indicated the occurrence of diverse tick species in different ecological settings [92,93,94]. Nidicolous *R. sanguineus* is mostly present in the peridomestic environment [75,76]. In addition to *R. sanguineus* infesting dogs and sometimes cats and foxes, *Dermacentor marginatus*, *D. reticulatus*, *Ixodes ricinus*, *Hyalomma marginatum*, *Haemaphysalis punctana* and *R. bursa* were collected in various ecological settings in Crimea [92,93,94]. In one location, 28% (n = 57) of *R. sanguineus* tested positive for *R. conorii* or *R. massiliae* [92]. A follow-up study tested 1972 ticks collected across the Peninsula, and 20.3% of 305 *R. sanguineus* tested positive via SFGR *glt*A screening PCR [93]. Subsequent sequencing identified DNA of *R. conorii* and *R. massiliae* in 36.4% and 9.2%, respectively, of PCR-positive brown dog ticks [93]. The same study detected *R. conorii* DNA in one *Hyalomma marginatum* [93]. Other ticks originating from different Crimean ecosystems included *H. punctata* (31.5% *glt*A positive, n = 1062), *H. marginatum* (34.5%, n = 139), *D. marginatus* (52.7%, n = 110), *D. reticulatus* (26.3%, n = 5) and *Ixodes ricinus* (13.9%, n = 47) [93]. SFGR prevalence ranged from the highest (50.6%) in ticks collected in the eastern steppe zone of the Peninsula to 12% in the west steppes, and only 4.5% in the mountain forest zone and southern coastal area [93]. Most importantly, six species of SFGR previously unknown in Crimea were identified, including *R. sibirica* subsp. *mongolotimonae*, *R. slovaca*, *R. aeschlimannii*, *R. monacensis*, *R. helvetica* and *R. raoultii* [93,94], all of which can cause human infection [7]. These pathogens had very focal distributions, suggesting they were restricted to specific habitats of their main animal hosts and/or established bird migration routes in Crimea [93]. These SFG rickettsiae cause varying human clinical responses; thus, some variations in clinical manifestations of local MSF cases may actually be due to infections with different *Rickettsia* species than *R. conorii conorii*. This can only be confirmed with molecular detection and identification of the SFG rickettsia agent causing each clinical rickettsial infection.

The distribution of MSF cases does not extend north of Crimea, despite the broad occurrence of brown dog ticks along Black Sea shores [72]. The most northern occurrence of *Rh. sanguineus* was documented at 48^o^N in Ukraine by collection of two females from a dog in a Kiev Park [72] and from grass in proximity to a railroad in the Kiev-Sviatoshynsky district [95]; however, these findings are considered to be incidental and may possibly have originated from a pet that had previously travelled to the endemic areas. There is also an unusual report of *R. conorii* DNA in one *Ixodes ricinus* [96]; however, this finding is based on a conserved fragment of an SFGR *glt*A gene and needs further confirmation.

*Rhipicephalus sanguineus* also occurs along the eastern shore of the Black Sea [97]; however, there are no data pertinent to the detection and identification of SFGR or reporting rickettsioses in these areas. An MSF outbreak was only once formally recognized and investigated in the City of Sukhumi in 1947; the *R. conorii* strain M-1 was isolated as part of that investigation [27,98], and later identified as a unique genotype of *R. conorii conorii* [99,100]. Nowadays, *R. conorii* is only rarely found in dog ticks in Georgia [101]. In contrast, surveillance of other species of questing ticks in these areas identified a similar repertoire of SFGR as those found in Crimea beyond *R. conorii*, including *R. helvetica*, *R. slovaca*, *R. monacensis*, *R. raoulti*, *R. aeschlimanii* and *R. massiliae*, with some regional variations depending on the tick species tested [95,101].

As stated in Section 3 above, MSF due to *R. conorii* has been formally diagnosed and confirmed using molecular methods in Turkey and *R. sanguineus* is identified among their human-biting ticks [35]; however, acarological studies conducted in the last 20 years are not consistent with the idea that MSF is a common illness in this country [102,103,104,105,106,107,108]. We identified only one case-report providing indirect evidence for the role of *R. sanguineus* in the transmission of *R. conorii* in Turkey [109]. Depending on the study, researchers have collected up to 12 different species of ticks parasitizing humans, domestic and wild animals and questing ticks from different areas of Turkey [102,103,104,105,106,107,108,110,111]; however, only a few collections included *R. sanguineus* [102,110,111]. These studies described the detection and identification of nine SFGR of known pathogenicity for humans and several SFGR with unknown virulence in different areas of Turkey, including Istanbul, Central Anatolia, the Yozgat province, Corum province and others [103,104,108,112]. Infrequent findings of *R. conorii* and some unusual tick associations may need further investigation and confirmatory study using a larger collection of ticks and simultaneous molecular speciation of ticks together with multigene characterization of the *Rickettsia* species detected. Molecular characterization of SFGR infections associated with *R. sanguineus*-infested domestic dogs will need to be performed to confirm the occurrence of a natural cycle of MSF in Turkey.

There are several old reports describing diverse hard ticks in Bulgaria and much work has dealt with Lyme disease and tick-borne encephalitis [113,114,115]. *Rhipicephalus sanguineus* is identified as the vector of *R. conorii* [32,33,116], and isolation of *R. conorii* has been reported (cited in [32]). Studies conducted in 1965–1967 reported 18.1% of *R. sanguineus* tested positive for *R. conorii*; this was probably established by inoculating laboratory animals with suspensions of homogenized ticks since the tick hemolymph test for rickettsial studies was not introduced until 1970 [117]. The most recent study summarized the results of testing 1780 specimens of six species of Ixodid ticks, including *R. sanguineus*, collected from regions endemic and non-endemic for MSF [32]. The average rate of positivity for SFGR was 22.8%, with no significant differences between infected ticks originating from MSF endemic (23.4%, n = 1052) and non-endemic (~22%, n = 780) areas, although the positivity rate for *R. sanguineus* was not reported as a separate variable [32,33,116]. A recent acarological investigation focused on ticks originating from the Strandja Nature Park from the Black Sea region of Bulgaria identified *R. monacensis* and *R. helvetica* in *I. ricinus* from vegetation, dogs and goats, and *R. aeschlimannii* in *Hy. anatolicum* (n = 1), *Hy. excavatum* (n = 2), *Hy. marginatum* (n = 4) and *Rhipicephalus* spp. (n = 1) from dogs and cattle. Only 10 *R. sanguineus* were collected from vegetation and they tested negative for SFGR, thus suggesting that *R. conorii* may have only a very focal presence among geographically dispersed populations of brown dog ticks in Bulgaria or may be associated with imported pets.

There are only infrequent findings of *R. conorii* in Romania, including its detection in 0.8% (n = 120) of *R. sanguineus* collected from owned dogs seen at a veterinary clinic in Bucharest [59] and in 1.9% (n = 53) of questing *H. punctata* from an urban site in Cluj-Napoca [118]. *Rh sanguineus* is found on dogs, cattle, sheep and wildlife [60,119]. Its typical habitat is within the southern lowlands of Romania, although *R. sanguineus* has been found in the Transylvania basin and northeastern parts of the country [119]. In recent years, it was also observed that the steppe tick *Rhipicephalus rossicus* is common and is the dominant species infesting dogs in the areas of southeastern Romania typically known as the classic habitat for the brown dog tick [120,121]. Pathogen carriage and the vectorial capacity of *R. rossicus* has not yet been determined for many tick-borne organisms; however, it should be noted that this tick may be commonly misidentified as *R. sanguinieus*, especially when engorged ticks are collected from dogs [89,119]. As for other ticks parasitizing humans, peridomestic and wildlife animals, their species diversity is similar to other countries situated in the Black Sea region. These ticks frequently tested positive for *R. helvetica* and *R. monacensis* (*Ixodes* spp.), *R. raoultii* and *R. slovaca* (*Dermacentor* spp.), *R. aeschlimanii* (*Hyalomma* spp.) and *R. massiliae* [58,118,122]; however, there are big variations in the reported SFGR positivity rate and prevalence of individual *Rickettsia* species detected depending upon the source of the ticks and molecular targets used.

## 9. Implications of Other *Rickettsia* and Rickettsioses Sympatric to *R. conorii conorii* and MSF in Black-Sea-Area Countries

An analysis of publications reporting acarological surveillance efforts in Crimea and neighboring territories indicates that endemic areas traditionally thought to be places for the occurrence and circulation of *R. conorii* and, thus, cases of MSF have been diagnosed in very diverse and complicated ecosystems with the co-circulation of multiple tick species carrying different SFGR [32,37,58,59,93,108]. Of course, *R. sanguineus* widely occupies a unique peridomestic niche both in rural and urban environments. However, there are multiple plausible scenarios of encroachment of these other natural habitats and ticks which can permit encounters with humans and their pet animals. At present, these systems probably exist in a condition close to their natural equilibrium with only infrequent contact with humans; however, numerous environmental and anthropogenic factors can contribute to their introduction to humans and their pets.

There are only a few reports of diagnosis of non-*R. conorii* infection in the countries surrounding the Black Sea; they are limited to PCR-diagnosed *R. sibirica mongolotimonae* and *R. slovaca* cases in Turkey [123,124], a serologically diagnosed *R. slovaca*-associated case of lymphadenopathy in Bulgaria [125] and *R. massiliae*, *R. slovaca* and *R. slovaca-R. raoultii* cases in Romania [126]. A serological IFA test using paired sera is the gold standard method for the diagnosis of SFG rickettsioses, and when used in conjunction with Western blotting analysis it may provide unequivocal identification of the etiological agent [44,52]; however, only a limited repertoire of antigens is commonly used for routine laboratory diagnosis in most countries. Clinical symptoms and a patient’s exposure history may provide additional clues to distinguish and diagnose other SFGR rickettsioses [Table 2]; however, these clues may be overlooked, particularly when physician familiarity is low [53]. These situations have several implications. Generic diagnosis of SFGR rickettsiosis without identifying its etiology may not make a significant difference from a clinical point of view and patient management; however, it will not contribute to accumulating specific information about the spectrum of SFGR responsible for human morbidity in each region. This situation may also negatively affect vector control programs since different SFGR are carried by different tick species with diverse habitats and animal host associations. On the other hand, when a case of MSF or another spotted fever group rickettsiosis is misdiagnosed as another illness, it may contribute to unnecessary morbidity and mortality among local residents [38,61,127,128]. The Black Sea coast is an important tourist destination for all the surrounding countries, and many travel-related cases may be overlooked [31].

As has been already mentioned above, some MSF cases in Turkey may be misdiagnosed as viral hemorrhagic fevers [52]. Moreover, there is a much longer list of clinical entities that may be confused with rickettsioses. These include upper respiratory tract infection, pneumonia, urinary tract infection, viral gastroenteritis, non-rickettsial bacterial sepsis, thrombotic thrombocytopenic purpura, idiopathic vasculitis and viral or bacterial meningoencephalitis [52]. Therefore, it takes an astute physician to distinguish these presentations and initiate correct empiric treatment to prevent poor patient outcomes. Systemic evaluation of patients’ symptoms, a detailed clinical picture and epidemiological clues together with family and travel history and known exposure and risk factors should be performed together with reviewing standard laboratory tests (total blood count, peripheral blood smear, blood chemistry and hepatic function panel) to implement a proper patient management plan. Doxycycline is the drug of choice for treatment of all tick-borne rickettsial diseases in patients of all ages, including children aged <8 years [16,52]. Prompt favorable response to it has been suggested as a useful clinical clue for rickettsioses. Clarithromycin and azithromycin can also be acceptable therapeutic alternatives to tetracyclines for children aged <8 years with Mediterranean spotted fever [152]; however, the same trial did not evaluate therapeutic effects on other SFG rickettsiae. Prophylactic treatment for rickettsial diseases in persons who have had recent tick bites and are not ill is not recommended [153]. It is also not recommended to prescribe antibiotics to asymptomatic individuals seropositive for SFGR regardless of their past treatment history [154].

A recent systematic review estimated that diagnosis of MSF based only on clinical symptoms misses 57.9% (6922/11,956) of the total patients presenting with an eschar and 81.6% (6922/8478) of patients when the eschar is absent or overlooked [155]. To ensure adequate patient management, proper laboratory confirmatory diagnosis is necessary, which is no longer challenging for SFG rickettsioses. Molecular methods can provide a definitive etiological diagnosis of rickettsiosis; however, clinical specimens must be collected within a very narrow period of time and prior to starting antibiotic therapy because the rickettsial DNA load in human blood depends on the *Rickettsia* species and severity of the illness [156,157]. Furthermore, rickettsial quantity in patients suffering from Rocky Mountain spotted fever appears to have a circadian rhythm, so the largest numbers are present during early morning hours [158]; however, similar observations for other rickettsioses are not available. Therefore, a skin biopsy of the eschar or skin rash is considered a more informative and reliable specimen for confirmatory diagnosis of rickettsioses [52,159]. Testing eschar scabs and eschar swabs is recommended to minimize invasive procedures associated with skin biopsies, which are not as acceptable for pediatric patients or for eschars on sensitive areas of the skin [160,161,162]. The utility of this approach has been demonstrated by confirming acute infections due to *R. conorii*, *R. slovaca* and *R. sibirica mongolotimae* [160,161].

The use of real-time PCR is now a common practice for detecting rickettsial DNA in clinical specimens. The use of multiplex PCR permits better specificity and accuracy of the diagnosis, especially when multiple etiological agents are suspected. A combination of broad-spectrum SYBR-Green PCR assay targeting the *Rickettsia* OmpB gene and *R. conorii*-specific *omp*A TaqMan was evaluated for differential diagnosis of MSF in endemic areas where other pathogenic SFGR are present [163]; the analytical sensitivity of both assays was <10 DNA copies. This approach was similar to another duplex PCR that combines pan-*Rickettsia* targets such as the 16S rRNA gene or 23S rRNA gene and group-specific *glt*A or another genus-specific gene like the hypothetical protein A1G_04230 to conduct differential detection of typhus group rickettsiae and *R. rickettsii*, respectively, and other *Rickettsia* species [164,165]. A reverse transcription PCR assay targeting the more stable and higher copy number rickettsial 23S rRNA gene permitted a 100-fold increase in analytical sensitivity compared with DNA-based detection methods and had comparable performance for testing banked patient samples [166]. However, a negative result cannot exclude clinical disease.

It is expected that the broader availability of nucleic acid-based methods and their use in regular clinical practice will improve routine diagnosis of spotted fever group rickettsioses and permit more effective treatment of these diseases in the region. Droplet digital PCR is one of the possible approaches and may be most useful for multiplex testing [167]. Next-generation sequencing provides powerful methodology permitting relatively quick identification of the etiological agent, especially for patients with atypical clinical manifestations and unclear epidemiologic evidence of a tick bite or exposure; however, this platform is not fully validated with rickettsioses and may pose challenges to complete identification when only a small number of *Rickettsia* reads are generated and other abundant microbial flora are present in the clinical sample [9,168]. Inexpensive Oxford Nanopore long-read technology can be used for genome sequencing of *Rickettsia* [169]. This less expensive technology is also proposed to be compatible with point-of-care diagnostics in resource-limited settings. Other commercial NGS-based methods may be useful for the detection of *Rickettsia* and diagnosis of rickettsioses [170]. Metagenomic sequencing of ticks permits one to identify a range of tick-borne pathogens present in a particular area, including known pathogens and those that have not been previously associated with human disease [171,172,173]

## 10. Conclusions and Highlights

MSF is an endemic disease for the Crimean Peninsula. For 85 years, it has been diagnosed annually along the Black Sea coast and manifests in sporadic cases or outbreak forms. MSF is a seasonal disease, with the majority of cases occurring in the spring and summer months during the period of peak tick activity. The incidence rate depends on the population of brown dog ticks, the number of abandoned and stray dogs and inconsistent animal and vector control measures. In recent years, moderate to severe clinical manifestations were the most common and presented with the classic clinical triad of fever, eschar and rash. Persons in all age groups are affected, but elderly people with underlying health conditions often have the most malignant course of the illness. The epidemiological, ecological and clinical features of MSF in Crimea are very similar to other regions in the Black Sea basin. The presence of diverse species of human-biting ticks in many eco-systems and the circulation of numerous species of *Rickettsia* known to cause spotted fever group rickettsioses with a wide range of clinical manifestations is challenging for both physicians and public health officials. An effort to utilize new rickettsial molecular diagnostic platforms and assays for routine clinical practice and vector surveys will provide a better basis for future improvements in treatment and a reduction in all of the spotted fever rickettsioses occurring in this region.

## Figures and Tables

**Table 1:** Clinical findings and symptoms of MSF in Crimea and other countries in the Black Sea Basin.

Symptom or Clinical Finding	MSF in Crimea		MSF in Bulgaria			MSF in Turkey		MSF in Romania
	Alymov 1939 & Andreev 1941 [21,22]	Gafarova 2004 [63]	Popivanova et al., 2007 [50]	Pishmisheva et al., 2014 ^a^ [48]	Baltadzhiev et al., 2020 ^b^ [47]	Mert et al., 2006 [36]	Kuloglu et al., 2012 [35]	Pitigoi et al., 2013 [31]
Number of cases observed	Unknown	137	774	257 (1253)	544	15	128	171
Incubation period	5–10	2–15 (5–7 average)	3–10< days	1–14 days (3.2 days average)	5–7 days	0–6 days	NR	
Acute onset	yes	yes	yes	yes	yes	yes	yes	yes
Fever	100% (39–40 °C)	100% (39–40 °C)	99.24%	98.8%	99.14–100%	100% (39.5–40 °C)	100%	99.4%
Fever (duration)	10–15 days	5–10 days	3–7< days	2.5 d (average)	NR	7–14 days	1–8 days	
Headache		83%	33.69%	18.7% (61.93%)	33.62–34.11%	87%	66.9%	43.1% (n = 151)
Myalgia (arthralgia)		54.7%	74.13%	40.08% (78.77%)	74.13–68.23%	93%	60.3%	43.4% (n = 152) ^d^
Malaise (adynamia)		50%		NR	68.31–54.11%	NR	NR	NR
Arthralgia		29.2%		NR		100%	NR	NR
Chills		yes	67.39%	NR	67.45–60%	100%	NR	NR
Eschar	72%	81%	77.04%	72.76% (67.2%)	76.93–73.68%	13%	70.3%	67.3%
Lymphadenitis	32%	23.3%		13.3%	Not observed	NR	NR	NR
Painful lymph nodes	16%	45%	Mostly absent	54.09% (10.93%)	39.2% ^a^	NR	NR	NR
Persisting pigmentation in the area of eschar and/or rash	2–3 years	2–3 months	Yes [after rash}	NR	NR	NR	NR	NR
Rash, onset	2–4 days	2–7< days	3–5 days	NR	3–5 days	2nd day of fever	12 h to 10 days	NR
Rash, duration, days	1–4 days	4–5 days		NR	NR	NR	NR	NR
Rash appearance	Polymorphic, maculopapular to papulo-petechial	Polymorphic: maculopapular (73%) to roseo-papular (23.3%)	Papular (49.9%), maculopapular (50.09%) and hemorrhagic 915.69%)	Maculopapular ^a^	Maculopapular	Maculopapular, also petechiae in 4 cases	Maculopapular and petechial	Maculopapular or purpuric
Rash, occurrence	80–100%	77.4%	99.27%	100% (98.2%)	100%	100%	95.3% (10.6%)	98.2%
Rash, location	Trunk to palm and soles	Trunk to face, palms and soles	Palms and soles, sometimes face and head	Face, palms and soles	Face, body trunk and limbs including palms and feet	Limbs and trunk (centripetal type)	Palms and soles	NR
Hypotension	36%	21.8%	NR	NR	NR	NR	NR	NR
Hepatomegaly	40%	42.3% (n = 58)	60.29%	83.27% (78.93%)	46.44–23.68%	13%	11.6%	NR
Splenomegaly	32%	2.2%	46.44%	Yes ^a1^	13.84–63.15% ^b^	13%	5.6%	NR
Euphoria	64%	NR	NR		NR	NR	NR	NR
Depression	16%	NR			NR	NR	NR	NR
Insomnia	80%	NR			NR	NR	NR	NR
Severity of disease								
Severe	12%	14.6%	33.85%	2.7%	29.5% ^a^	0%	10.9%	0%
Average|Moderate severity	20%	83.2%	56.68%	97.3%	41.16–32.79	100%	89.1%	100%
Decreased appetite (anorexia)		58.3%	57.35%	NR	57.32–70.58%	NR	NR	NR
Nausea (vomiting)		14.4%	11.29% (17.66%)	NR	11.2 (17.67) 52.94 (50.98)%	NR	NR	NR
Abdominal pain	+	+	6.73%	NR	6.68–23.52%	NR	NR	NR
Diarrhea	+	+	1.82%	NR	1.72–5.88%	NR	NR	NR
Sore throat	No	No	0.36%	NR	0.43–8.23%	20% ^c^	NR	16.8% (n = 149) ^d^
Conjunctivitis	NR	16% (n = 20)	5.1%	NR		20%	19.8%	
Laboratory findings:								
Leukopenia	+	+	3.86%	NR	NR ^b^	13%	12.7%	NR
Monocytosis	+	+	NR	NR	NR ^b^	NR	NR	NR
Leucocytosis	+	11.3%	9.62%	NR	NR ^b^	74%	32%	31.8% (n = 170)
Thrombocytopemia	_	11.03%	↓ most cases	NR	NR ^b^	33%	52.5%	50.9% (n = 159)
Erythrocyte precipitation rate	↑, 72%	84.5% (n = 137)		NR	NR ^b^	↑, 100% (n = 7)	NR	↑, 55.1% (n = 138)
↑ C-reactive protein				NR			98%	NR
Proteinuria	32%	29.9%		NR		NR	NR	NR
Elevation of liver enzymes (AST and/or ALT)		AST: 34.3% ALT: 72.7%	58.1% (54.46%)	>50% ^a1^		60%	72%	78.5% (n = 158)
Fatality	<2%	None	9.44% [adults]	0%	3.5% (4.1%) ^b^	None	1.6%	0%

^a^ This study reports observations of 257 children and 1253 adults, with most details provided for pediatric patients; one pediatric patient presented with necrotic rash. It is stated that splenomegaly most occurred in children and that more than half of the cases had increased aminotransferase values; however, exact prevalence is not reported. First number corresponds to observations in pediatric patients, and second numbers are observations in adults [48]. ^b^ This study by Baltadzhiev et al. [47] reports observations of MSF in 464 adults and 85 children; table includes data for both age groups, respectively. Enlarged and painful lymph nodes were observed only in pediatric patients. Indicates observations of combined enlarged liver and spleen. Briefly, 29.5% includes severe (88 cases) and malignant (55) forms and 19 lethal cases. Fatality was calculated per total number of cases and for adults only (in brackets). Details of laboratory parameters are not reported; however, it is indicated that they were consistent with MSF. ^c^ Authors also reported hyperemia of tonsils and pharynx in 4 (27%) patients [36]. ^d^ Arthralgia was identified in 23.7% (n = 152) and reported as a separate symptom. Reported respiratory symptoms occurred in 16.8% of 149 patients examined; however, specific clinical manifestations are not described [31].

**Table 2:** Clinical signs and symptoms of rickettsial diseases caused by *Rickettsia* species sympatric to *R. conorii conorii* in the Black Sea region ^a^.

Feature or Symptom	Etiological Agent					
	*R. raoultii*	*R. slovaca*	*R. aeschlimanii*	*R. monacensis*	*R. massiliae*	*R. sibirica mongolotimonae*
Number of cases ^b^	72	143	10	12	9	56
Case geography	France, Slovakia, Poland, Russia, China, Romania, Spain	France, Slovakia, Italy, Germany, Hungary, Spain, Poland, Romania, Russia	South Africa, Greece, Algeria, Italy, Morocco, Russia, China	Spain, Italy, Portugal, Netherlands, South Korea, China	Italy, France, Romania, Greece, Tunisia, Argentina	France, Spain, Portugal, Greece, Egypt, Algeria, Sri Lanka, Turkey
Primary tick vector	*Dermacentor* sp.	*Dermacentor* sp.	*Hyalomma* sp.	*Ixodes* sp.	*Rhipicephalus* sp.	*Hyalomma* sp., *Rhipicephalus* sp.
Prevalence in ticks ^c^	1.8–58%	1.7–24.3%	0.8–77.2%	0.5–57%	2–92%	4–8%
Fever	37% (n = 70)	21.8% (n = 133)	80% (39–40 °C)	100% (38–40 °C)	100%	100%
Eschar	20.3% (n = 69)	40.5% (n = 116)	80%	75% (n = 8)	100%	94.6%
Rash	5.6%	8% (n = 124)	60%	87.5% (n = 8)	80%	73%
Rash, type	Not specified in reports	Frequently not specified in reports	Maculopapular	Macular, maculopapular or erythematous	Maculopapular to purpuric rash	Maculopapular
Headache	25%	53% ^d^	50%	75% (n = 8)	44%	Reported very rarely
Lymphadenopathy	32.9% (n = 70)	60.8% (n = 120)	NR	50% (n = 8)	22%	57.7% (n = 26)
Lymphangitis	NR	NR	NR	NR	NR	37.5%
Complications, atypical symptoms	Pulmonary edema, lethargy,	Alopecia, asthenia, cellulitis of the face	Acute hepatitis, arthritis, retina hemorrhage	NR	Acute vision loss, seizure, myalgia	Encephalitis, myopericarditis, retinal vasculitis
Co-infection	Tick-borne encephalitis virus, *B. miyamotoi*, Tacheng tick virus, *R. slovaca*	*Coxiella burnetii*, *R. raoultii*	*B. burgdorferi*	*B. burgdorferi*, *O. tsutsugamushi*	NR	NR
References	[129,130,131,132,133]	[7,126,129,130,131,134,135]	[130,136,137,138,139]	[140,141,142,143]	[144]	[123,145,146,147,148,149]

^a^ Clinical signs and symptoms are summarized based on clinical case reports published in English language peer-reviewed journals. Cases due to *R. helvetica* were not included due to relatively mild, self-limited illness and only a very limited number of molecularly confirmed clinical cases in the peer-reviewed literature [7,143,150,151]. NR, not reported. ^b^ Patient number is based on clinical case reports and reports of molecular testing of clinical samples without detailed clinical history. ^c^ The prevalence in ticks varies based on the geographic region, individual study design and characteristics of the tick collections, and tools used for laboratory testing and may be different from the numbers reported herein. ^d^ Reported according to the summary presented in [7].

## Data Availability

The data presented in this study are available on request from the corresponding author.
